# Función de los profesionales de la salud de rectificar la información errónea que tienen los pacientes más allá de corregir los hechos^*^

**DOI:** 10.26633/RPSP.2021.60

**Published:** 2021-05-18

**Authors:** Brian G. Southwell, Jamie L. Wood, Ann Marie Navar

**Affiliations:** 1 Facultad de Medicina de la Universidad Duke Durham Estados Unidos de América Facultad de Medicina de la Universidad Duke, Durham, Estados Unidos de América.; 2 Centro de Ciencias de la Comunicación, RTI International Research Triangle Park Estados Unidos de América Centro de Ciencias de la Comunicación, RTI International, Research Triangle Park, Estados Unidos de América.

La mayoría de los pacientes confían en los profesionales de la salud (1), pero muchos también recurren a fuentes fuera del consultorio para obtener información médica. Si bien muchos recursos proporcionan información correcta (por ejemplo, organismos gubernamentales de salud, organizaciones profesionales y grupos de defensa del paciente), no toda la información que los pacientes encuentran lo es. Los pacientes pueden encontrar información médica errónea procedente de una variedad de fuentes en línea, lo que pueden tener consecuencias importantes para la salud.

Los prestadores de atención de salud pueden desempeñar un papel esencial para rectificar la información médica errónea, pero aún no han tenido la oportunidad de hacerlo plenamente. (En algunas disciplinas se han logrado avances, como en el caso de los pediatras en la mitigación de la información errónea sobre las vacunas.) Rectificar eficazmente la información errónea requiere más que intentos de simplemente desacreditar las percepciones equivocadas. Cuando los médicos se encuentran ante pacientes que tienen información errónea, tienen la oportunidad de conocer sus valores, preferencias, comprensión y fuentes de información. La capacitación sistemática de los profesionales de la salud para que rectifiquen con empatía y curiosidad la información errónea adquirida por los pacientes, reconociendo las limitaciones de tiempo y recursos, será una contribución esencial para la mitigación futura de la información médica errónea.

## EXPOSICIÓN DEL PACIENTE A LA INFORMACIÓN ERRÓNEA

A pesar de algunas iniciativas recientes de las plataformas de medios sociales para reducir o contrarrestar la información médica errónea (por ejemplo, https://bit.ly/3f4vBeE), los pacientes pueden encontrar una amplia gama de información médica inexacta en línea con un esfuerzo mínimo. Se puede encontrar información errónea en algunos sitios web que promocionan o venden productos alternativos “naturales” y publican materiales al respecto (2). Se puede encontrar información errónea en algunas publicaciones en los medios sociales o artículos escritos descuidadamente en diversos sitios. Una variedad de información errónea sobre remedios, causas y políticas acompañó la llegada de la pandemia de la enfermedad por el coronavirus del 2019. Al mismo tiempo, las posibles consecuencias de la información médica errónea también varían. Las afirmaciones inexactas que llegan a grandes públicos y alientan a las personas a participar en comportamientos nocivos son diferentes de las afirmaciones técnicamente incorrectas, pero relativamente intrascendentes (3).

A pesar del acuerdo en torno a la idea de que existe información errónea problemática, los pacientes y prestadores también afrontan retos para caracterizar de manera confiable la información de salud de calidad alta y baja. Se podría intentar juzgar la información evaluando la calidad científica de la investigación notificada, la transparencia respecto del patrocinio de la investigación y la medida en que se describen las limitaciones de la investigación (Véase https://bit.ly/2D74fqY, donde se presentan ejemplos de preguntas que pueden plantearse). Sin embargo, aplicar eficazmente ese enfoque de tipo “lista de verificación” requiere una comprensión científica de entrada que va más allá de lo que se puede esperar de la mayoría de los pacientes. Este tipo de preguntas sirven mejor como motivación para que el paciente consulte con un profesional de atención de salud, y no como instrumento independiente para que lo utilicen los pacientes.

## MITIGAR LOS EFECTOS DE LA INFORMACIÓN ERRÓNEA

Debemos mejorar la relación de los pacientes con los profesionales de la salud, lo que significa que necesitamos instrumentos y enfoques para mejorar los distintos tipos de conversaciones entre pacientes y profesionales sobre afirmaciones médicas inexactas. Aquí podemos aprender de una categoría específica de esfuerzos en este sentido: los esfuerzos emprendidos para disipar la reticencia de los pacientes respecto de las vacunas. Leask et al. (4) elaboraron una guía para que los profesionales de la salud la tengan en cuenta cuando respondan a las inquietudes de los padres respecto de la vacunación. Hacen hincapié en una postura que ofrece a los padres asistencia en la toma de decisiones en lugar de tratar de persuadirlos directamente o de desacreditar fuentes de información específicas. En este enfoque se prioriza ofrecer asesoramiento informado sobre cómo pensar acerca de las decisiones sobre las vacunas en lugar de desacreditar fuentes de información específicas. Es importante destacar que también sabemos que, en algunos casos, incluso los propios profesionales de la salud pueden ofrecer información inexacta (5). Leask et al. (4) señalan la oportunidad que tienen los profesionales de la salud de averiguar las inquietudes que tienen los padres en estos encuentros y de reconocer, escuchar y mostrarse empáticos con ellos, al tiempo que señalan fuentes de información apropiadas. Sobre la base de ese tipo de enfoque, en la Universidad Duke y con el apoyo de la ABIM Foundation y Craig Newmark Philanthropies, hemos elaborado capacitación para que los médicos rectifiquen la información errónea, en la que se hace énfasis en mostrar empatía y escuchar, y se reconocen a la vez las limitaciones de tiempo.

Desarrollar la capacidad de escuchar inquietudes, preferencias y valores, así como de estar atentos a los entornos de información disponibles para detectar afirmaciones inexactas, requiere esfuerzo. Si bien algunos han pedido que las organizaciones médicas se encarguen de comprobar los hechos y responder en los medios sociales con miras a rectificar la información médica errónea, será esencial invertir en esfuerzos ampliables para establecer relaciones individuales con los pacientes. Consideremos, por ejemplo, la experiencia de las actividades de los Centros para el Control y la Prevención de Enfermedades de Estados Unidos para hacer el seguimiento de los viajeros que llegaban a Estados Unidos durante el brote del ébola del 2014 al 2015 (Para obtener más información, consulte https://bit.ly/2CAM2lJ.). La evidencia indica que un factor clave en la intención de los viajeros de cumplir los requisitos era la confianza, es decir, la medida en que los viajeros confiaban en el personal del programa con el que hablaban en un aeropuerto estadounidense sobre el seguimiento. Es más probable que haya confianza interpersonal en situaciones en las que las personas se encuentran directamente con un profesional de salud en persona (al menos virtualmente) que en situaciones en las que recibe información de otra manera. La confianza implica una relación interpersonal y no solo hechos.

Para participar en una conversación (sobre información o sobre lo que podría ser información errónea), los pacientes necesitan sentirse empoderados para plantear una idea que su prestador de atención de salud podría percibir como controvertida o problemática. En lugar de esperar que los pacientes planteen inquietudes sin que se les pida que lo hagan, los prestadores de atención de salud deberían invitar a sus pacientes a que inicien conversaciones sobre posible información errónea. Por ejemplo, invitar a los pacientes a que comuniquen lo que podría estar afectando sus decisiones sobre su tratamiento con una pregunta abierta (p. ej., “¿Qué ha oído o sabe ya acerca de su tratamiento o enfermedad?”) podría abrir un espacio de conversación útil.

Entender la información errónea como una fuerza en la vida de un paciente también requiere una evaluación del propio contexto en el que este vive. A menudo, las experiencias de los pacientes o de sus amigos y familiares afectan a la forma en que ellos se relacionan con la información médica. Si un paciente tiene un familiar que ha sufrido las consecuencias de un error médico, esto puede hacer que confíe menos en el sistema de atención de salud en general y que sea más propenso a creer la información errónea centrada en los “peligros” de los tratamientos tradicionales. Algunos pacientes pueden confiar menos en el sistema de atención de salud y los médicos debido a las desigualdades de salud y al maltrato histórico (6). Las creencias religiosas o espirituales también pueden afectar a las creencias de los pacientes respecto de su cuerpo y a las decisiones relativas a las opciones terapéuticas, desde elegir alternativas naturales hasta rechazar tratamientos.

Los prestadores deben reconocer que los pacientes seguirán recurriendo a internet, a compañeros y a familiares para obtener asesoramiento médico. Es poco probable que se obtengan resultados positivos al disuadir por completo a los pacientes de recurrir a fuentes alternativas. Mitigar los efectos de la información errónea requiere que los prestadores habiliten a los pacientes con fuentes precisas de información para que satisfagan sus propias necesidades de autoeducación. El material educativo de los pacientes debe incluir información sobre recursos fiables.

Si bien la investigación sobre los efectos de la información médica errónea está aumentando, sabemos relativamente poco acerca de la manera de rectificarla mediante la intervención clínica. Así como la investigación ha ayudado a determinar técnicas óptimas para la toma de decisiones compartida entre pacientes y prestadores, necesitamos una base de evidencia elaborada sistemáticamente para rectificar la información errónea en un entorno clínico.

## CAMINO A SEGUIR

Los profesionales de atención de salud pueden abordar situaciones en las que los pacientes tienen información médica errónea aprovechando las oportunidades para escucharlos, prestar atención a los entornos de información electrónica existentes y guiar a los pacientes hacia una mejor comprensión de la evidencia médica sometida a arbitraje, posiblemente en coordinación con iniciativas dirigidas a mejorar los conocimientos básicos en materia de noticias e información (7). Hacerlo implicará más que emitir pronunciamientos correctivos sobre falacias. Los profesionales de la salud tendrán que invertir tiempo para entender la descripción que los pacientes hacen de la información errónea y la forma en que la valoran, y trabajar en cooperación con ellos a fin de priorizar las fuentes creíbles.

## Declaración.

Las opiniones expresadas en este manuscrito son responsabilidad del autor y no reflejan necesariamente los criterios ni la política de la *RPSP/PAJPH* y/o de la OPS.
